# Impulsivity and Attention in Obsessive Compulsive and Tic Disorders: Mismatch in Self-Report and Behavioural Data

**DOI:** 10.3390/jcm12062277

**Published:** 2023-03-15

**Authors:** Leonard Guenter Koenn, Sina Kohl, Sophia Schleyken, Jens Kuhn

**Affiliations:** 1 Department of Psychiatry and Psychotherapy, University Hospital Cologne, University of Cologne, 50923 Cologne, Germany; 2 Department of Psychiatry, Psychotherapy and Psychosomatic Medicine, Johanniter Hospital Oberhausen, 46145 Oberhausen, Germany

**Keywords:** obsessive-compulsive disorder, OCD, Tourette syndrome, Tourette’s syndrome, impulsivity, impulsive behaviour, self-reported, self-assessed, Barratt Impulsiveness Scale-11, BIS-11, immediate then delayed memory task, IMT/DMT, attention

## Abstract

Impulsivity is a multidimensional, cross-diagnostic behavioural construct that has been described in various psychiatric disorders including obsessive-compulsive disorder (OCD) and Tourette syndrome (TS). Different interpretations of results in the past have raised the question of heightened impulsivity as an explanatory model for self-described impulsive behaviour, especially in OCD. Our study included 16 patients with OCD, 14 patients with TS, and 28 healthy control subjects (HC). Self-assessed impulsivity was examined by the Barratt Impulsiveness Scale-11 (BIS-11), and the behavioural test used was the immediate and delayed memory task (IMT/DMT). Significantly heightened self-assessed impulsivity of the patient collective compared to HC could be observed in in only one dimension: lack of attention (χ^2^ (2) = 24.910, *p* < 0.001). Post-hoc tests were performed using Bonferroni adjusted alpha levels of 0.0167 per test (0.05/3) and revealed significantly higher scores in patients with OCD (*M* = 19.57, *SD* = 2.82), *z* = 4.292, *p* < 0.001 as with TS (*M* = 19.38, *SD* = 3.62), *z* = 3.832, *p* < 0.001 compared to HC (*M* = 13.78, *SD* = 3.18). In patients with OCD, correlations between the dimension of obsessive thoughts with a lack of attention in the form of first-order factor cognitive instability could be shown (*n* = 14, *p* = 0.024, *r_s_* = 0.599) while in patients with TS, tic symptomatology correlated significantly with second-order factor attentional impulsivity (*n* = 12, *p* = 0.027, *r_s_* = 0.635). In behavioural testing, no significant group differences could be observed either in impulsive behaviour (IMT: χ^2^ (2) = 4.709, *p* = 0.824; DMT: χ^2^ (2) = 0.126, *p* = 0.939) or in sustained attention (IMT: χ^2^ (2) = 0.388, *p* = 0.095; DMT: χ^2^ (2) = 0.663, *p* = 0.718). Heightened impulsivity as an explanatory model for the observed lack of attention, especially in patients with OCD, should be questioned and interpretation biases considered in the future. The necessity of a multidimensional approach to the research of impulsivity is underscored by our results.

## 1. Introduction

In day-to-day life, we are confronted with a myriad of stimuli that influence our decisions and behaviour. Inhibitory control mechanisms allow us to act flexibly and consider ourselves in complex situations. Impaired inhibitory control mechanisms often entail pathological impulsivity [1]. Impulsivity represents a complex multidimensional behavioural construct with varying definitions (e.g., tending to act thoughtlessly and inconsiderately; an insensitivity to consequences; the preference of a smaller, immediate, as opposed to a larger reward at a later point of time; or the inability to suppress motor impulses) [2,3,4]. Usually, pathological impulsivity has negative consequences for the affected person or their environment [5]. In everyday life, these consequences can be as heterogenous as its dimensions: deficits in communicating with fellow human beings without being able to control your emotions [6] (e.g., an outburst of anger towards colleagues). A rash, unpredictable action (e.g., risky driving behaviours [7]) can lead to health or financial consequences for those acting impulsively as well as for others, problematic Internet use, nicotine smoking, or excessive use of alcohol [8]. Impulsivity is also discussed as a risk factor for non-suicidal self-injuries [9] and seems to be an important factor of lower quality of life in different psychiatric disorders [10,11].

Negative consequences of a lack of attention as a dimension of impulsivity include problems in staying concentrated and not being distracted from an important task [12] (e.g., writing an important term paper or when giving a presentation). Impulsivity in the form of careless actions may represent overlooking a component of a cooking recipe with moderate consequence, but when crossing a road inattentively, it can become dangerous.

It must be mentioned that the focus of research on impulsive behaviour has been on its negative aspects. However impulsive behaviour can also be helpful and advantageous in some situations, particularly when a fast and spontaneous reaction is essential [13] (e.g., by seizing the moment). Impulsive decision making can prevent thinking circumstances apart and thus missing opportunities. This type of impulsivity is called functional impulsivity [14]. An important example from an evolutionary point of view could be an impulsive fight or flight behaviour as a response to an unpredictable attack [15]. 

It has become clear that impulsivity is a diverse construct with advantageous as well as disadvantageous aspects and multiple dimensions. Depending on the focus of the investigation of past work regarding effect and dimensionality, various test methods have been created by factor analysis and used in studies including self-assessment as well as behavioural examinations [12,16,17,18,19,20,21].

Impulsivity has been observed in various psychiatric disorders [22]. Patients with bipolar disorder, for example, show more inattentive behaviour [23] and the extent of impulsive behaviour seems to correlate with poorer outcome and heightened suicidality in bipolar disorder [24]. In patients with substance use disorder, it has been described as a risk factor for the age of onset, treatment outcome, and relapse risk in various types of self-assessed and behavioural tests [25,26,27]. In patients with borderline personality disorders, impulsivity is considered as a key feature with a wide range of clinical manifestations (e.g., avoiding the feeling of emptiness through impulsive actions or self-harm). Impulsivity is argued to be a crucial target in psychotherapeutical treatment [28]. 

Attention plays a central role in the study of behaviour and is a relevant cross-diagnostic factor. While it can be seen as an independent, multi-layered construct with different dimensions and definitions [29], a lack of attention is also described in the context of impulsive behaviour [12]. This study focused on the research of impulsive behaviour in patients with obsessive compulsive disorder (OCD) and patients with Tourette syndrome (TS) in the most-used questionnaire, the Barratt Impulsiveness Scale-11 (BIS-11). The questionnaire meets the dimensional approach of impulsivity and includes deficits of attention, which was of particular interest to us as there has been ongoing discussion in the past regarding its interpretation. Attentional deficits have been observed in patients with OCD in the past, and authors have drawn different conclusions regarding heightened impulsivity as an explanatory model. This was due to evidence of non-increased impulsive behaviour in the other dimensions of motor and non-planning as well as evidence of possible confounding factors such as obsessive thoughts [30,31,32]. Patients with TS have also been assessed for impulsive behaviour using the BIS-11 in the past. However, due to the different hypotheses to be tested, multidimensionality was not always considered [33,34], or if so, a direct comparison between TS and healthy control subjects was not made [30,35].

It has become clear that due to the diverse influence of impulsivity on our everyday behaviour and the resulting consequences in dealing with ourselves and our fellow human beings, it is of great interest in research. This study seeks to better understand the subtle distinctions and overlaps of its multifaceted constructs and dimensions by comparing patients with OCD, patients with TS, and healthy control subjects (HC). A central feature of this study is the detailed consideration of each of the dimensions of impulsive behaviour, the comparison between the patient collective and HC as well as the use of an additional behavioural test in the form of the Immediate and Delayed Memory Task (IMT/DMT). 

## 2. Materials and Methods

Patients were recruited from the interdisciplinary outpatient clinic for obsessive-compulsive spectrum disorders at University Hospital Cologne. Handouts and information about the study were placed in the waiting area. If patients showed interest in the study, scientific staff, not involved in the treatment of the patients, would provide them with further information. All participants provided written informed consent for the procedure and participation in the study. The study was approved by the Ethics Committee of the University Hospital Cologne. The recruitment of HC was conducted by telephone contact with persons in a database who had already participated in previous studies at the Department of Psychiatry and Psychotherapy and had consented to further contact for study purposes. The study included 16 patients with OCD, 14 patients with TS, and 28 HC. Patients were pre-diagnosed in our outpatient clinic based on a semi-structured clinical interview by an experienced clinician. In our study protocol, pre-diagnosed OCD patients were tested with the Obsessive Compulsive Inventory-Revised (OCI-R) and patients with pre-diagnosed TS with the Adult Tic Questionnaire (ATQ) to evaluate the current severity of the respective symptoms. The OCI-R is a theoretically driven and brief self-report measure for the assessment of obsessive-compulsive symptoms [36] and captures the full range of OCD symptoms with excellent psychometric properties [37]. Healthy control participants had screenings for mental disorders before as they had participated in previous studies. In our study, they were tested with the OCI-R and the ATQ to screen for subclinical obsessive-compulsive symptoms or tics. 

The subjects completed a behavioural test as well as various questionnaires that were performed in randomised order. The behavioural test used was the IMT/DMT. It is a continuous performance test to study sustained attention, working memory, and impulsivity [21,38]. The response of the subjects to target-directed and non-target-directed stimuli was distinguished, which differed only marginally. The more frequent the response to target-directed stimuli (correct responses), the more attentive the patient. The more frequent the response to non-target stimuli (commission errors), the more impulsive the patient’s behaviour [39,40]. The Barratt Impulsiveness Scale-11 (BIS-11) is the most widely used questionnaire to assess impulsivity by self-assessment and contains 30 items in total [12]. Each of these items is assigned to one of six different first-order factor dimensions. Two first-order factor dimensions in turn form one second-order factor dimension, leaving three second-order factor dimensions (attentional, motor, and non-planning impulsivity) in total (see Table 1). 

The OCI-R is an 18-item questionnaire for the self-assessment of obsessive-compulsive symptoms that are divided into six dimensions and a total score [41]. The ATQ is designed to examine tic symptoms by self-assessment. Tic symptoms are divided into 14 items for motor and 13 items for phonic tics [42]. The Beck Depression Inventory-II (BDI-II) is one of the most commonly used questionnaires to assess depressive symptomatology and includes 21 items [43]. The Wender Utah Rating Scale (WURS) retrospectively allows for the assessment of the severity of attention deficit hyperactivity disorder (ADHD) in an adult’s childhood. The German-language short form (WURS-K) was used in this study and contains 25 items [44]. All statistical analyses were performed with commercially available statistical software (IBM SPSS, version 26). The collected data were subdivided into patients with OCD, patients with TS and HC based on the diagnoses, and the resulting group assignment. The Kolmogorov–Smirnov test did not show a normal distribution in all parameters of the data used (see Table A1 and Table A2). This necessitated the use of nonparametric statistical procedures. First, a descriptive review of the collected data was performed. Then, to test for differences in the central tendencies between the groups, the Kruskal–Wallis test was performed. If the results of the Kruskal–Wallis test were significant, additional post-hoc tests were performed using Bonferroni adjusted alpha levels of 0.0167 per test (0.05/3). In order to draw conclusions about the correlations between the collected data of different tests, the Spearman rank correlation coefficient r_s_ was also determined. According to Cohen, correlations of r_s_ <0.3 are considered weak, r_s_ = 0.3–0.5 are moderately strong, and r_s_ >0.5 are strong [45]. The significance level was defined as 5%.

## 3. Results

### 3.1. Patient Collective

#### Demographic Data

Included in the study were 16 patients with OCD (10 male, six female) with a mean age of 36.31 years (±13.65), 14 patients with TS (11 male, three female) with a mean age of 28.79 (±4.89), and 28 HC with a mean age of 38.11 (±13.84).

### 3.2. Reliability Analysis

Cronbach′s α for the BIS-11 total score was 0.77 in the overall sample and ranged from 0.72 to 0.81 across the three groups (OCD, TS, and HC). Examining the ATQ Cronbach’s α in patients with TS was 0.89 for the total score, 0.79for motor tics, and 0.83 for vocal tics. Patients with OCD showed Cronbach’s α in the OCI-R total score of 0.87 and ranged from 0.76 to 0.98 in the subscales. The WURS-K showed a Cronbach’s α of 0.75 in patients with TS, 0.85 in patients with OCD, and 0.79 in HC. Cronbach’s α for the BDI-2 total score was 0.88 in patients with TS, 0.95 in patients with OCD, and 0.75 in HC. 

### 3.3. BIS-11

#### 3.3.1. Kruskal–Wallis Test

Results from the Kruskal-Wallis Test are shown in Table 2. 

#### 3.3.2. Bonferroni Post-Hoc Test

Results from the Bonferroni Post-Hoc Test are shown in Table 3. 

### 3.4. IMT/DMT

#### Kruskal–Wallis Test

In the evaluation of the Correct Responses objectifying sustained attention, no significant group differences in either the IMT (χ^2^ (2) = 0.388, *p* = 0.095) or the DMT (χ^2^ (2) = 0.663, *p* = 0.718) were found. Commission Errors account for impulsivity and showed no significant group differences in either the IMT (χ^2^ (2) = 4.709, *p* = 0.824) or the DMT (χ^2^ (2) = 0.126, *p* = 0.939). The Commission Errors/Correct Responses ratio, also used for the measurement of impulsivity, did not show significant group differences in either the IMT (χ^2^ (2) = 0.411, *p* = 0.814) or the DMT (χ^2^ (2) = 0.474, *p* = 0.789). 

### 3.5. Correlations

#### 3.5.1. Patients with TS

In patients with TS, tic severity correlated strongly positively in the ATQ total score with the second-order factor attentional impulsivity of the BIS-11 questionnaire (*n* = 12, *p* = 0.027, r_s_ = 0.635).

#### 3.5.2. Patients with OCD

In patients with OCD, the symptom dimension obsessive thoughts of the OCI-R correlated strongly positively with the dimension cognitive instability (first-order factor) of the BIS-11 (*n* = 14, *p* = 0.024, r_s_ = 0.599). Moreover, the symptom dimension controlling of the OCI-R showed a strong positive correlation with the dimension cognitive instability (first-order factor) of the BIS-11 (*n* = 14, *p* = 0.001, r_s_ = 0.758).

### 3.6. Comorbidities

Depressive symptoms were examined using the BDI-II (for results, see Table A4). ADHD symptoms in childhood and adolescence were recorded by the WURS-K (for results, see Table A5). Statistical analysis regarding the group differences in the severity of depressive or ADHD symptoms were conducted (Table A6 and Table A7). No correlations were performed to test for a possible influence of underlying comorbidities on impulsive behaviours; this was also discussed in the limitations of the study.

## 4. Discussion

The complexity of impulsivity underlines the meaningfulness of its detailed consideration. In our study, the inclusion of different research approaches using self-assessment and behavioural testing and the multidimensional approach in their evaluations proved revealing. Patients with OCD as well as patients with TS showed significant deficits in attention via self-assessment. Our data revealed a direct correlation: the stronger the obsessive-compulsive or tic symptoms, the less attentive the patients were. To be able to classify these results in the best possible way, it is interesting to observe that other dimensions of self-assessed impulsivity (motor and non-planning) remained without significant differences compared to HC. Different causes of this influence on attention are conceivable, and according to our results, an increased impulsivity, especially in patients with OCD, as the sole explanatory model is not obvious. Moreover, a lack of attention could not be objectified in patients either with OCD or in patients with TS.

Tic symptomatology correlated with the phenomenon of a lack of attention in the form of focus on current tasks (first-order factor attention), and as a result, with poorer second-order factor attentional impulsivity. A comparison with the current literature is difficult as the studies concerning the self-assessment of impulsivity in patients with TS by means of the BIS-11 or its evaluation are expandable. For example, the studies of Atkinson-Clement et al. showed a more impulsive behaviour in patients with TS compared to HC [33], while Delorme et al. found no significant differences [34], but none of the studies considered the individual dimensions beyond the total score of the questionnaire. Thus, a specific conclusion regarding a lack of attention in the context of impulsive behaviour cannot be drawn. Further studies did justice to the multidimensional approach and examined clinical pictures such as patients with OCD and comorbid tic disorders [30] or addictive disorders, which additionally included a group of patients with TS [35], however, the results were not tested for significant differences between patients with TS and HC, as this was not the focus of these studies. Including different test methods, the evidence for reduced sustained attention (the capacity to maintain attention over a period of time, to react to desired stimuli, and to ignore faulty ones [46]), in childhood patients with TS is predominant, especially in the case of comorbid ADHD [47,48,49,50]. In adulthood, however, the data are less clear: in some cases, reduced attention could be demonstrated [51,52], in other studies, however, not [53,54]. Overall, the field of research of attention deficits in patients with TS in adulthood is expandable and is handled differently by different authors in the evaluation of the results. Our study underlines the assumption that multidimensionality should be considered when evaluating the BIS-11. Our results do not indicate a cross-dimensionally heightened impulsivity; more studies in the future with larger cohorts would be necessary to investigate this hypothesis further.

The existing literature examining impulsive behaviour in patients with OCD using the BIS-11 is more extensive. Increased scores in the cross-dimensional total score could be shown, while individual dimensions were also considered separately. The dimension that was decisive for the significant differences was usually attentional impulsivity [55,56,57,58]. The dimensions motor and non-planning often remained without significant group differences [30,31,58,59]. Stein et al. even found lower motor impulsivity in OCD patients compared to HC in a large cohort [60]. In congruence with these results, we found increased impulsivity in patients with OCD compared to HC in the dimension of a lack of attention; remarkably, in all first- and second-order factors, there was a poorer ability to concentrate on current tasks as well as increased racing thoughts and thought jumps. Resulting from this, an overall heightened attentional impulsivity was found. No significant group differences in the dimension of motor and non-planning or the total scores could be shown (see Table 2).

The fact that other dimensions (motor and non-planning) often remain without significant results has already caused discussion in the past. Some authors argue that an elevated score in a questionnaire measuring impulsivity is directly linked to increased impulsive behaviour [57,58]. However, other authors have questioned this interpretation and state that increased scores in the total score need to be interpreted with caution if attentional impulsivity is the only dimension with a stronger expression. At this point, increased cross-dimensional impulsive behaviour in patients with OCD is still a matter of debate [32,61].

Given the above consideration, it is highly interesting to discuss hypothesis of the relation between OCD and lack of attention. Interestingly, our data showed a correlation where the more pronounced the obsessive-compulsive symptoms (controlling and obsessive thoughts), the more frequently patients with OCD reported a lack of attention (in form of first-order factor cognitive instability, e.g., racing thoughts and thought jumps). Similar to our findings, past studies have found a direct correlation between obsessive-compulsive symptoms and decreased attention [30,31,55]. As a result, a lack of attention has been discussed as a consequence of increased obsessive thoughts and not necessarily in the context of increased impulsivity [30,31]. Obsessive thoughts are often fearful and worrying, and patients try unsuccessfully to resist these intrusions. Therefore, patients with OCD in our study reported difficulties in resisting “unpleasant” and “repulsive” thoughts and in “controlling their thoughts” in the OCI-R questionnaire. Accordingly, it would be conceivable that the simultaneously observable lack of attention in the BIS-11 (e.g., in the scoring of the statements “I concentrate easily” or “I have extraneous thoughts when thinking”) arose due to the obsessive-compulsive symptomatology in the form of uncontrollable intrusions and not necessarily as an independent phenomenon in the context of increased impulsivity. 

Summerfeldt et al. took this consideration one step further [30]. The authors argued that patients with OCD would not necessarily have reduced attentional impulsivity despite elevated scores on the BIS-11. It would be possible that the wording chosen to describe impulsive behaviour could be understood, at least in part, as describing obsessive thoughts. The subjects would thus confirm that while thinking “incidental thoughts come into their mind” or “thoughts race through their head”, referring perhaps, even exclusively, to their existing obsessive thoughts. Hence, whether patients refer to a disorder immanent phenomenon (intrusive thoughts) or to their process of thinking in general cannot be uncovered by the BIS-11. 

**Figure 1:**
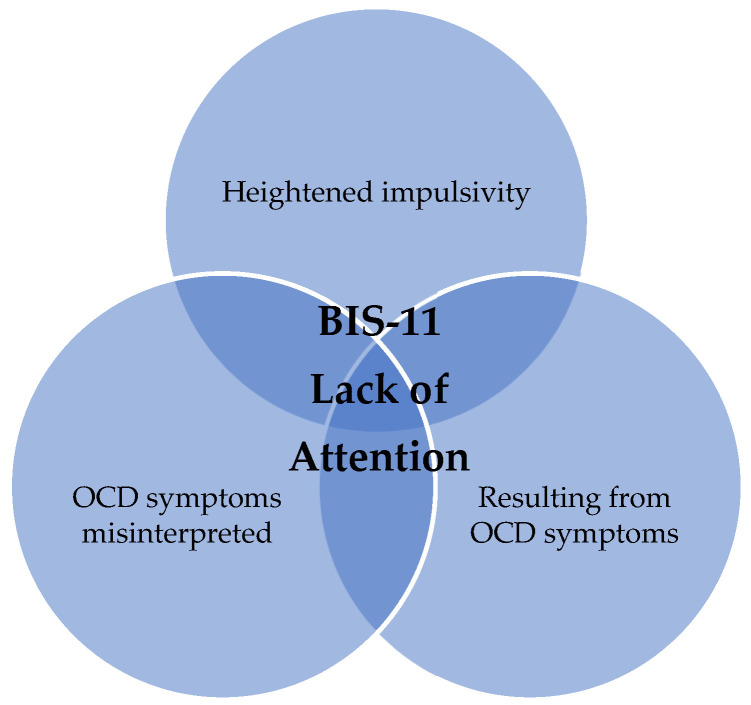
Conceivable explanatory model for a self-reported lack of attention in the BIS-11 questionnaire in patients with OCD.

In summary, the assessment of an increased total score in the BIS-11 must always be considered in relation to the dimensions. Impulsivity could still be a reason for the described lack of attention, but it is not obvious as the sole explanatory model based on our results and in the synopsis with the described previous literature. An alternative conceivable explanatory model according to our results as well as previous studies is shown in Figure 1. 

In this context, the evaluation of objectifiable attention is exciting. As described, attention can present itself in different ways. The IMT/DMT, as a behavioural test, captures “sustained attention”: the capacity to maintain attention over a period of time, to react to desired stimuli, and to ignore faulty ones [46]. In the BIS-11, a lack of attention is captured in the context of impulsive behaviour (e.g., in the scoring of the statements “I don’t pay attention”, “I can concentrate easily”, or “I am a persistent thinker.”). Despite the different perspectives on the lack of attention of the two test options, overlaps can be identified. Whilst the BIS-11 patients with OCD showed poorer attention, the results of the IMT/DMT remained without significant group differences. One explanatory approach would support the hypothesis that the self-perceived decreased attention in the BIS-11 might have been obsessive thoughts misinterpreted. Despite the overlap of the definitions of attention in IMT/DMT and BIS-11, they are, of course, not equivalent, and the inference cannot be drawn without bias. For example, different parameters are used to capture sustained attention and impulsivity in the IMT/DMT [21]. Even if overlaps of sustained attention with the dimension of impulsivity of the BIS-11 are assumed, it remains an explanatory possibility that different results could also be due to the type of testing. A large study by Cyders et al. showed that although self-perceived cognitive impulsivity overlaps with impulsivity at the behavioural level, these two test methods appear to examine different constructs [62]. Congruent to this, in our study, subjective impulsivity examined by self-assessment questionnaires showed no correlation with impulsivity objectifiable by behavioural testing in patients with OCD (see Table A3). In fact, there are even indications that subjective approaches by self-assessment show a higher reliability than objective ones by behavioural testing [63]. The results of the IMT/DMT would therefore not exclude an actual lack of attention in patients with OCD.

Just as complex and multi-layered as the behavioural phenomena under consideration are the individuals studied with their respective diagnoses and clinical manifestations. Despite the small group of patients, the frequent occurrence and degree of expression of depressive symptoms [64,65,66,67] (see Table A4) as comorbid ADHD [68,69,70,71] (see Table A5) could be seen in our patient collective. Thus 80% of the patients with OCD and 46.2% of patients with Tourette’s syndrome suffered from depressive syndromes of different degrees, and in each case, showed significantly more pronounced depressive symptoms than HC (see Table A6 and Table A7). A total of 46.7% of patients with OCD and 53.8% of patients with TS could be diagnosed with ADHD in retrospect. Patients with TS showed significantly more pronounced ADHD symptoms in childhood and adolescence in the Kruskal–Wallis test and post-hoc analysis compared to HC (see Table A8 and Table A9). 

Depressiveness [72,73,74] as well as ADHD [75,76,77] have shown potential influences of the observed behaviours in the past, and this circumstance should be considered in the assessment of our results as we did not examine the correlations between the underlying comorbid symptomatology and therefore its possible influence on the observed impulsive behaviour. Future studies should not only describe the occurrence of, but also its influence on impulsiveness. It would also be of great interest to also use further diagnostic groups that suffer from impulsivity.

Symptom severities of obsessive-compulsive symptomatology in patients with OCD and tic symptomatology in patients with TS can be found in Table A10 and Table A11. Results from screening HC for obsessive compulsive or tic symptoms can be found in Table A12 and Table A13.

Other limitations of the study that should be mentioned are the small patient collective and the heterogeneity in the severity of the different diseases and their treatment status.

In summary, our results suggest that multidimensionality should always be considered when examining impulsivity. Even when a study design does not allow for various types of testing, the dimensional evaluation of single tests like the BIS-11 can provide relevant information about impulsive behaviours regarding its different aspects. In our study, the question of heightened impulsivity in patients with OCD as well as TS would have been denied, without further consideration of the dimensional construct, missing to discuss the self-reported lack of attention. Therefore, our study underscores the necessity of a detailed interpretation of the various measuring tools for impulsive behaviour with its overlaps and subtle differences regarding their different parameters. Impulsivity represents an important building block in our understanding of behaviour beyond defined psychiatric diagnoses. Despite the information already acquired, there is often still no consensus regarding its exact dimensional classification [78,79,80,81]. Further studies with larger cohorts are therefore of great importance for a detailed understanding of this behavioural phenomenon.

## Figures and Tables

**Table 1:** Factor structure of the BIS-11 questionnaire according to Patton et al. [12].

Second-Order Factor	First-Order Factor	Description
Attentional Impulsivity	Attention	Focusing on current task
	Cognitive Instability	Intruding thoughts
Motor Impulsivity	Motor	Acting quickly
	Perseverance	Stable lifestyle
Non-planning Impulsivity	Self-Control	Enjoys mental challenges
	Cognitive Complexity	Plans and thinks deliberatively

**Table 2:** Testing for significant group differences in the second-order factors attentional, motor, and non-planning impulsivity and the total score of the Barratt Impulsiveness Scale-11 using the Kruskal–Wallis Test. Abbreviations: df, degree of freedom; *p*, asymptotic significance; χ^2^, chi-square.

Kruskal–Wallis	Attentional	Motor	Non-Planning	Total
χ^2^	24.910	5.79	1.054	4.825
df	2	2	2	2
*p*	<0.001	0.056	0.590	0.090

**Table 3:** Th post-hoc Bonferroni test of the first-order factors attention and cognitive insatiability (belonging to the second-order factor attentional impulsivity) of the BIS-11 questionnaire. Abbreviations: Adj., Adjusted; HC, healthy control subjects; OCD, obsessive-compulsive disorder; Sig., Significance; Std., Standard; TS, Tourette syndrome.

Group 1–Group 2	BIS-11 First-Order Factor	Test Statistic	Std. Error	Std. Test Statistic	Sig.	Adj. Sig.
HC–TS	Attention	20.283	5.294	3.832	<0.001	<0.001
	Cognitive Instability	16.819	5.222	3.221	0.001	0.004
HC–OCD	Attention	22.165	5.164	4.292	<0.001	<0.001
	Cognitive Instability	19.882	5.095	3.902	<0.001	<0.001
TS–OCD	Attention	−1.882	6.040	−0.312	0.755	1.000
	Cognitive Instability	−3.063	5.959	−0.514	0.607	1.000

## Data Availability

The data presented in this study are available on reasonable request from the corresponding author. The data are not publicly available due to missing consent.

## Appendix A

**Table A1:** Testing for parametric distribution in the BIS-11 using the Kolmogorov–Smirnov test with significance correction according to Lillefors. Abbreviations: df, degrees of freedom; *p*, significance.

Dimension	Statistic	df	*p*
First-order factors			
Attention	0.143	54	0.008
Cognitive Instability	0.124	54	0.038
Motor	0.119	54	0.053
Perseverance	0.166	54	<0.001
Self-Control	0.122	54	0.044
Cognitive Complexity	0.107	54	0.187
Second-order factors			
Attentional	0.076	54	0.200
Motor	0.129	54	0.025
Non-planning	0.091	54	0.200
Total Score	0.078	54	0.200

**Table A2:** Testing for parametric distribution in the IMT/DMT using the Kolmogorov–Smirnov test with significance correction according to Lillefors. Abbreviations: CE, commission errors; CR, correct responses; df, degrees of freedom; *p*, significance.

Parameters	Statistic	df	*p*
Correct Responses			
IMT	0.101	53	0.200
DMT	0.166	53	0.001
Commission Errors			
IMT	0.104	53	0.200
DMT	0.088	53	0.200
CE/CR			
IMT	0.121	53	0.049
DMT	0.066	53	0.200

**Table A3:** Testing for Spearman correlations between dimensions of the BIS-11 questionnaire and IMT/DMT in patients with OCD (*n* = 13). Abbreviations: Com., commission errors; Corr., correct responses; DMT, delayed memory task; IMT, immediate memory task; *p*, significance (2-sided); *r_s_*, Spearman correlation coefficient.

BIS-11 Dimension	Spearman	IMT Corr.	DMT Corr.	IMT Com.	DMT Com.	IMT Com./Cor.	DMT Com./Cor.
Attention first-order factor	*r_s_*	0.309	0.141	0.069	−0.182	0.039	−0.157
	*p*	0.304	0.646	0.823	0.551	0.900	0.607
Cognitive Instability first-order factor	*r_s_*	−0.041	−0.108	0.070	0.000	0.076	−0.015
	*p*	0.895	0.726	0.821	1.000	0.806	0.962
Motor first-order factor	*r_s_*	−0.202	0.163	−0.028	0.036	0.064	0.080
	*p*	0.509	0.595	0.929	0.907	0.837	0.795
Perseverance first-order factor	*r_s_*	0.179	0.065	−0.080	−0.344	−0.213	−0.324
	*p*	0.558	0.832	0.796	0.250	0.484	0.280
Self-Control first-order factor	*r_s_*	−0.260	0.050	−0.202	−0.155	−0.113	−0.105
	*p*	0.391	0.872	0.508	0.613	0.712	0.733
Cognitive Complexity first-order factor	*r_s_*	0.017	0.394	−0.305	−0.508	−0.293	−0.531
	*p*	0.957	0.183	0.312	0.076	0.331	0.062
Attentional second-order factor	*r_s_*	0.241	0.069	0.202	−0.022	0.194	0.003
	*p*	0.428	0.822	0.508	0.943	0.526	0.993
Motor Second-order factor	*r_s_*	−0.199	0.180	−0.058	−0.119	−0.006	−0.061
	*p*	0.515	0.557	0.851	0.699	0.986	0.844
Non-planning second-order factor	*r_s_*	−0.099	0.226	−0.253	−0.399	−0.226	−0.369
	*p*	0.747	0.458	0.403	0.176	0.458	0.214
Total Score	*r_s_*	−0.181	0.110	−0.038	−0.192	−0.016	−0.137
	*p*	0.553	0.721	0.901	0.529	0.957	0.655

**Table A4:** Scores in the Beck Depression Inventory-II (BDI-II) in groups. Abbreviations: HC, healthy control subjects; IQR, interquartile range; M, mean; Max, maximum; Mdn, median; Min, minimum; OCD, obsessive-compulsive disorder; SD standard deviation; TS, Tourette syndrome.

Group	*n*	M	SD	Mdn	IQR	Min–Max
TS	13	10.31	7.353	10	4.5–14	0–28
OCD	15	14.38	13.912	26	18–33	2–59
HC	27	3.15	3.382	2	1–6	0–15

**Table A5:** Scores in the German short version of the Wender Utah Rating Scale (WURS-K) in groups. Abbreviations: HC, healthy control subjects; IQR, interquartile range; M, mean; Max, maximum; Mdn, median; Min, minimum; OCD, obsessive-compulsive disorder; SD standard deviation; TS, Tourette syndrome.

Group	*n*	M	SD	Mdn	IQR	Min–Max
TS	13	33.69	11.814	33.00	26–40	17–61
OCD	15	28.13	13.912	28.00	15–40	10–51
HC	27	23.26	10.208	20.00	15–29	7–47

**Table A6:** Testing for significant group differences in the Beck Depression Inventory-II (BDI-II) using the Kruskal–Wallis test. Abbreviations: BDI-II, Beck Depressions Inventory-II; df, degree of freedom; *p*, asymptotic significance; χ^2^, chi-square.

Kruskal–Wallis	BDI-II
χ^2^	28.431
df	2
*p*	<0.001

**Table A7:** Testing for significant group differences using the Bonferroni post-hoc test in the Beck Depression Inventory-II (BDI-II). Abbreviations: Adj. Sig., adjusted significance; HC, healthy control subjects; OCD, obsessive-compulsive disorder; *p*, asymptotic significance; Sig., significance; Std., standard; TS, Tourette syndrome.

Group 1–Group 2	Test Statistic	Std. Error	Std. Test Statistic	Sig.	Adj. Sig.
HC–TS	14.756	5.393	2.736	0.006	0.019
HC–OCD	26.933	5.145	5.235	<0.001	<0.001
TS–OCD	−12.177	6.054	−2.011	0.044	0.133

**Table A8:** Testing for significant group differences in the Wender Utah Rating Scale-Kurzform (WURS-K) using the Kruskal–Wallis test. Abbreviations: WURS-K, Wender Utah Rating Scale-Kurzform; df, degree of freedom; *p*, asymptotic significance; χ^2^, chi-square.

Kruskal–Wallis	WURS-K
χ^2^	6.133
df	2
*p*	0.047

**Table A9:** Testing for significant group differences using the Bonferroni post-hoc test in the Wender Utah Rating Scale-Kurzform (WURS-K). Abbreviations: Adj. Sig., adjusted significance; HC, healthy control subjects; OCD, obsessive-compulsive disorder; *p*, asymptotic significance; Sig., significance; Std., standard; TS, Tourette syndrome.

Group 1–Group 2	Test Statistic	Std. Error	Std. Test Statistic	Sig.	Adj. Sig.
HC–TS	13.281	5.401	2.459	0.014	0.042
HC–OCD	5.737	5.152	1.114	0.265	0.796
OCD–TS	7.544	6.062	1.244	0.213	0.640

**Table A10:** Scores in the Adult Tic Questionnaire (ATQ) of patients with TS (*n* = 12). Abbreviations: IQR, interquartile range; M, mean; Max, maximum; Mdn, median; Min, minimum; SD, standard deviation.

Dimension	M	SD	Mdn	IQR	Min–Max
Motor	36.00	17.63	31.00	26.75–47.00	9–76
Phonic	20.58	18.42	14.50	10–21.25	6–66
Total	56.58	34.03	45.50	39–68.5	15–142

**Table A11:** Scores in the Obsessive Compulsive Inventory-Revised (OCI-R) of patients with OCD (*n* = 15). Abbreviations: IQR, interquartile range; M, mean; Max, maximum; Mdn, median; Min, minimum; SD, standard deviation.

Dimension	M	SD	Mdn	IQR	Min–Max
Washing	4.53	4.45	2.00	0–9	0–12
Controlling	7.27	3.56	6.00	6–12	1–12
Ordering	5.47	4.63	6.00	0–9	0–12
Obsessive Thoughts	8.80	3.32	11.00	5–12	3–12
Hoarding	3.20	2.46	3.00	0–6	0–7
Neutralising	4.00	4.00	3.00	0–7	0–11

**Table A12:** Scores in the Obsessive Compulsive Inventory-Revised (OCI-R) of healthy control subjects (*n* = 27). Abbreviations: IQR, interquartile range; M, mean; Max, maximum; Mdn, median; Min, minimum; SD, standard deviation.

Dimension	M	SD	Mdn	IQR	Min–Max
Washing	0.93	1.429	0.00	0–1	0–5
Controlling	1.59	1.803	1.00	0–2	1–6
Ordering	2.63	2.436	2.00	1–4	0–9
Obsessive Thoughts	0.85	1.460	0.00	0–1	0–7
Hoarding	1.67	1.330	1.00	1–3	0–4
Neutralising	0.41	0.888	0.00	0–1	0–4

**Table A13:** Scores in the Adult Tic Questionnaire (ATQ) of healthy control subjects (*n* = 28). Abbreviations: IQR, interquartile range; M, mean; Max, maximum; Mdn, median; Min, Minimum; SD, standard deviation.

Dimension	M	SD	Mdn	IQR	Min–Max
Motor	0.18	0.945	0.00	0–0	0–5
Phonic	0.00	0.00	0.00	0–0	0–0
Total	0.18	0.945	0.00	0–0	0–5
